# Supplementary material for: The role of Smarcad1 in retroviral repression in mouse embryonic stem cells
Source: Mob DNA. 2024 Mar 11;15:4. doi: 10.1186/s13100-024-00314-z (PMC10929159; doi:10.1186/s13100-024-00314-z)
Supplement: Supplementary file 1 — Supplementary Material 1. [file 13100_2024_314_MOESM1_ESM.pdf]

Supplementary materials:

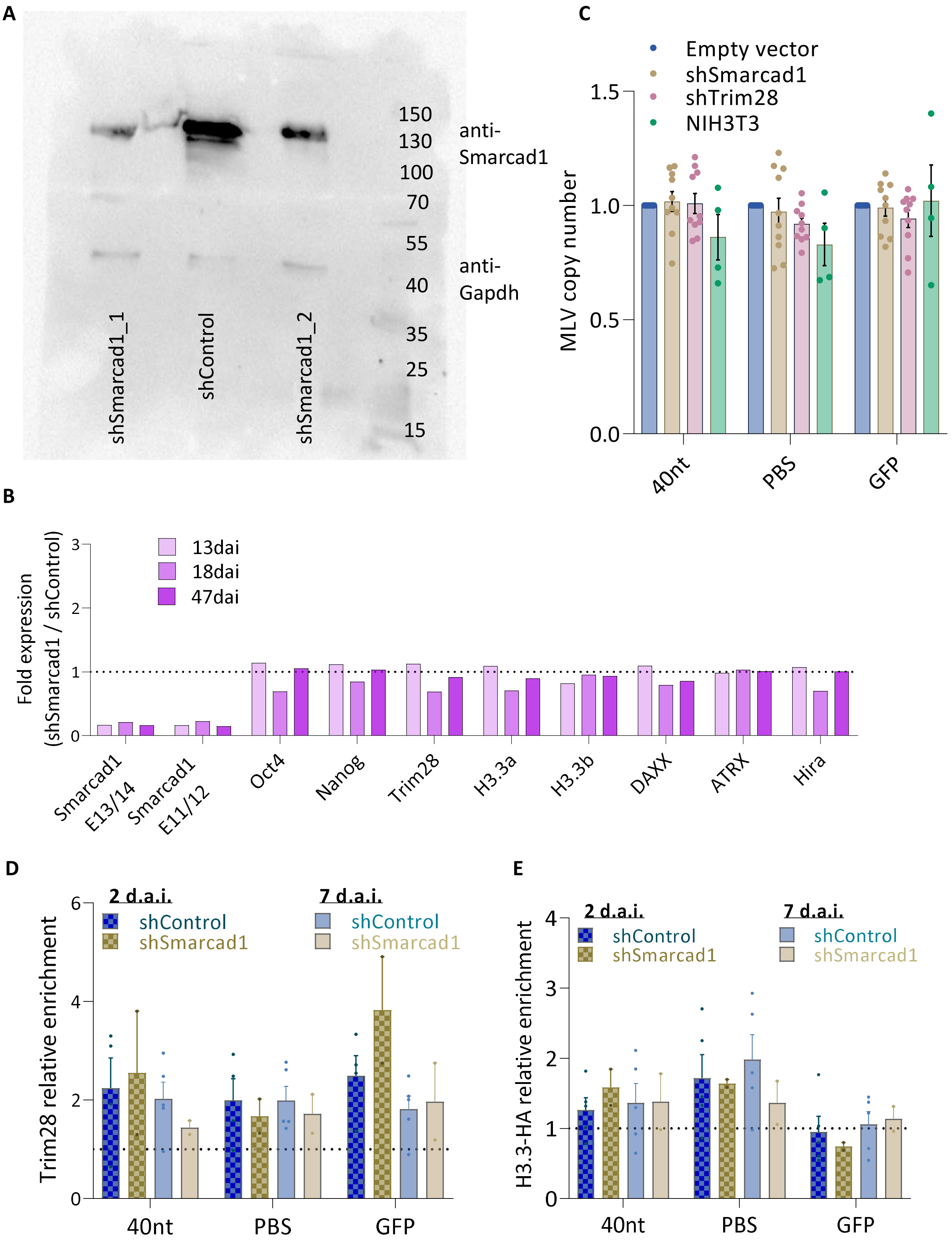

Supplementary Figure S1: Smarcad1 is recruited to repress incoming retroviral infection. (A) Immunoblotting of shSmarcad1 and shControl ESC extract using Smarcad1 antibody, with anti Gapdh as a loading control. A full blot is shown. (B) Expression levels over time following Smarcad1 depletion were measured by RT–qPCR (C). The calibration of viral copy numbers was determined by genomic DNA extraction and qPCR analysis using provirus primers on the provirus and normalized to Gapdh and control samples with one integration event. In two different experiments, the copy number was similar between the WT and Smarcad1-depleted cells. n=10, Data are the mean ± s.e.m. (D) ChIP–qPCR was performed after MLV-PBSgln infection using Trim28 antibody and (F) HA antibody at days 2 and 7 after infection (n=2). Values shown are mean ± s.e.m, relative to the total input samples and normalized to the signal of negative control (Gapdh or Hbb, respectively).

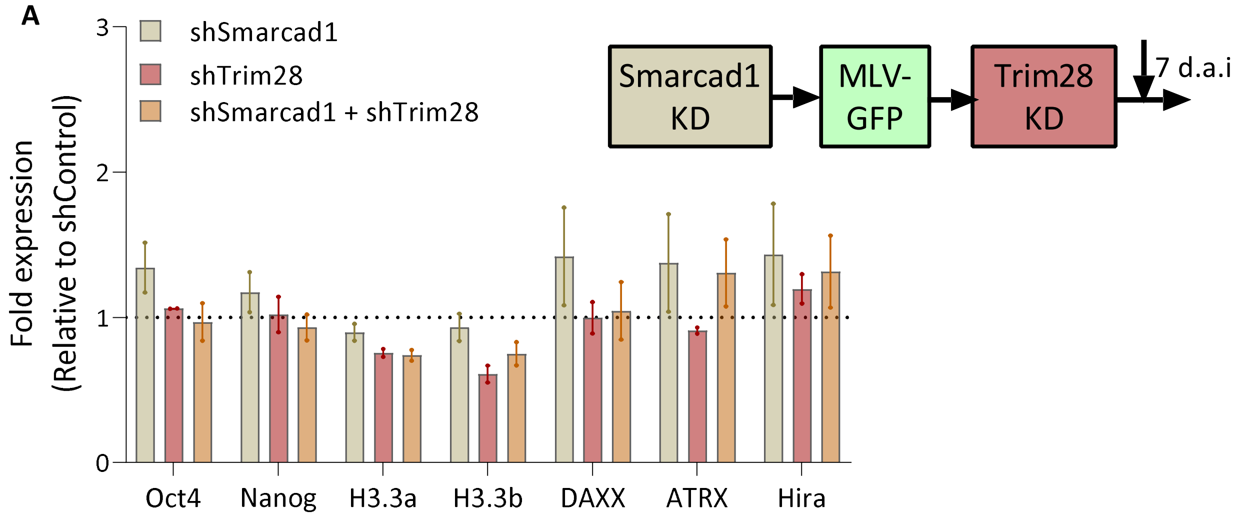

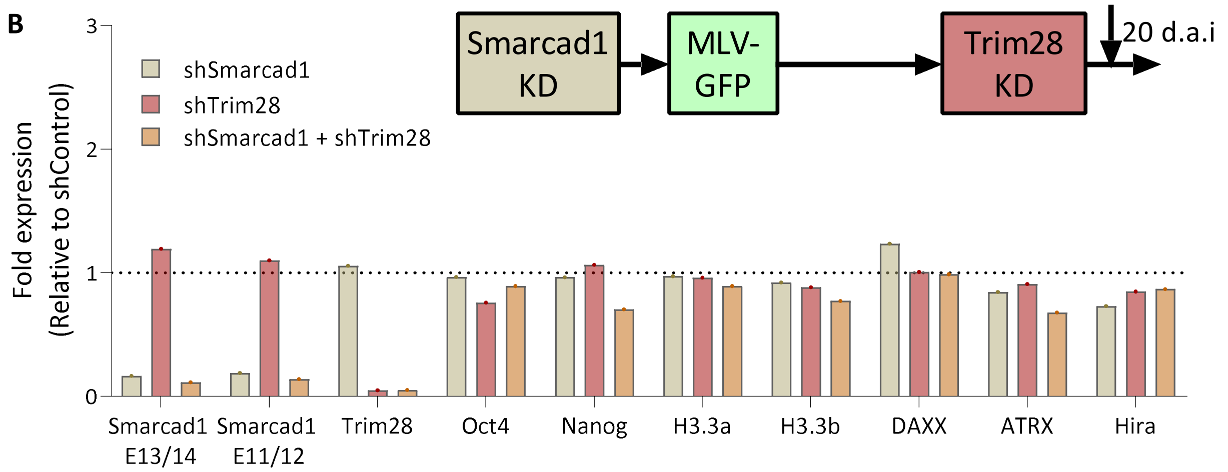

Supplementary figure S2: Smarcad1 and Trim28 depletion has an additive effect on MLV upregulation. (A) Expression levels of selected ERVs following Smarcad1 and Trim28 depletion were measured by RT–qPCR. Data are the mean ± s.e.m. (n=2-5) (B) Expression levels of key pluripotency genes and other factors involved in retroviral silencing following Smarcad1 and Trim28 depletion 20 d.a.i. were measured by RT–qPCR. Data are the mean ± s.e.m. (n=2-5). In all panels, the P value was calculated using a two-tailed unpaired Student's t test, *P<0.05, **P<0.01.

Supplementary Table S1: Primers list

Primer name	Target	5'-> 3' Sequence	Purpose in study
**GAPDH_F**	GAPDH	ACCTTTAGCCTTGCCCTTT	ChIP
**GAPGH_R**	(Genomic)	ACATCACCCCCATCACTCAT	
**m-Oct4_pr20**	Oct4	CACCGGACACCTCACAAAC	ChIP
**M-Oct4_pr19**	(Genomic)	TCTCCAGAGGATGGCTGAGT	
**Nanog36_F**	Nanog	ACACCTACGAGGCATTATTAAGCTAATTCC	ChIP
**Nanog36_R**	(Genomic)	GCCCATAAATGATTACAACAGCAGACTAA	
**L-mChip-Polrmt**	Polrmt	AGACACCTGCTGCCCTATGT	ChIP
**R-mChip-polrmt**	(Genomic)	GCTCCATCCCAGTGCTTTAC	
**Fkbp6_F**	FKbp6	CATGCTCGCTGCGTCTATC	ChIP
**Fkbp6_R**	(Genomic)	ATCTTGCCGCACAACTGTCT	
**L-mChip-Peg13**	Peg13	AGCCTCTGTGCTAGCGTCTC	ChIP
**R-mChip-Peg13**	(Genomic)	GGATACCTTCGAGCGTTGAG	
**MMERVGLN_F**	MMERVGLN	CGTAAGGACCCTAGTGGCTG	ChIP/qPCR
**MMERVGLN_R**	(Class I ERV)	GCACTCACTCTTCTTCACTCTG	
**VL30_U3I_LTR _F**	VL30 U3I LTR	AGATGTATTGCCAAACACAGG	ChIP/qPCR
**VL30_U3I_LTR_R**	(Class I ERV)	AGGGGGAATGGGGAGGGAA	
**RLTR1b_F**	RLTR1b	TCCTTCCCTTTGCCCTATTT	ChIP/qPCR
**RLTR1b_R**	(Class I ERV)	GGCTGGAACTGGTGAGATGT	
**MTA_F**	MTA	ATGTCTTGGGGAGGACTGTG	ChIP/qPCR
**MTA_R**	(Class II ERV)	AGCCCCAGCTAACCAGAACT	
**ETnERV2_F**	EtnERV2	ACAAATTCAGTATGGGCATC	ChIP/qPCR
**ETnERV2_R**	(Class II ERV)	GGGTACTGTTAAGACCCACA	
**IAPd-F**	IAP-D	TGAGAGAGGAGCGATCCCAA	ChIP/qPCR
**IAPd-R**	(Class II ERV)	GACTGTCGGCTATGCTCTCC	
**IAP1-MM_I-F**	IAP1 MM I	CGTTGCCTTGAGTTTCAACA	ChIP/qPCR
**IAP1-MM_I-R**	(Class II ERV)	GTCACATCCATCTGCCACAC	
**IAP5-Mm_I-F**	IAP5 MM I	GTGCGGTCAGTCCTACGTAA	ChIP/qPCR
**IAP5-Mm_I-R**	(Class II ERV)	CCCGGAGCAGAAGTGAAAGT	
**IAPEY3_i-F**	IAPEY3i	TGGTGTGGAAGATGCTGACC	ChIP/qPCR
**IAPEY3_i-R**	(Class II ERV)	CCCATCTCTGTTCGCCCATT	
**IAPLTR1a_I_MM-F**	IAPLTR1a	CAGTGCGCAGACTCATTCAT	ChIP/qPCR
**IAPLTR1a_I_MM-R**	(Class II ERV)	CTCGGCTCCTTCAAAGACTG	
**RLTR45_F**	RLTR45	TGCTTTTCCGACATGGTAAT	ChIP/qPCR
**RLTR45_R**	(Class II ERV)	AGTAACCCTGACCTGCTCCT	
**IAPEY5_I-F**	IAPEY5i	GACAATGGGCCAGCTTATGT	ChIP/qPCR
**IAPEY5_I-R**	(Class II ERV)	GCCTTGTCCTTGTGGATTGT	
**IAPEz_F_ChIP**	IAPEz	GCTCCTGAAGATGTAAGCAATAAAG	ChIP/qPCR
**IAPEz_R_ChIP**	(Class II ERV)	CTTCCTTGCGCCAGTCCCGAG	
**IAPEz-gag_F**	IAPEz gag	CACGCTCCGGTAGAATACTTACAAAT	ChIP
**IAPEz-gag_R**	(Class II ERV)	CCTGTCTAACTGCACCAAGGTAAAAT	
**IAPEz-gag_F**	IAPEz gag	CACGCTCCGGTAGAATACTTACAAAT	qPCR
**IAPEz-gag_R**	(Class II ERV)	CCTGTCTAACTGCACCAAGGTAAAAT	
**IAP gag F**	IAP gag	AATCTCAGAACCGCTCCATGA	ChIP/qPCR
**IAP gag R**	(Class II ERV)	TTTCTTAAAATGCCCAGGCTTT	
**IAP-5UTR F**	IAP 5UTR	CGGGTCGCGGTAATAAAGGT	ChIP/qPCR
**IAP-5UTR R**	(Class II ERV)	ACTCTCGTTCCCCAGCTGAA	
**MMERVK-10C_F**	MERVK10C	CAAATAGCCCTACCATATGTCAG	ChIP
**MMERVK R**	(Class II ERV)	GTATACTTTCTTCTTCAGGTCCAC	
**MERVL-F**	MERVL	ATCTCCTGGCACCTGGTATG	ChIP/qPCR
**MERVL-R**	(Class III ERV)	AGAAGAAGGCATTTGCCAGA	
**ORR1_F**	ORR1	CTTTAGTTGATGGCCCAGGA	qPCR
**ORR1_R**	(Class III ERV)	CCAACTCTGCCCTCTGTAGC	
**Line1_1 ChIP_F**	L1-Md family	GATTACCAGATGGCGAAAGG	ChIP/qPCR
**Line1_ChIP_R**	(Non-LTR)	AGTGCTGCGTTCTGATGATG	
**PBS_F**	MLV PBS	TGGCCAGCAACTTATCTGTG	ChIP
**PBS_R**	(Provirus)	CAGGCGCATAAAATCAGTCA	

**GFP2-F**	MLV GFP	AGCTGAAGGGCATCGACTT	ChIP
**GFP2-R**	(Provirus)	GATGCCGTTCTTCTGCTTGT	
**MLV +4-F**	MLV TSS	CAGTCCTCCGATTGACTGAG	ChIP
**MLV +91-R**	(Provirus)	GGAACAGCGAGACCACAAGT	
**40nt_L**	MLV 40nt	CCTCCACACACCATCATCCA	ChIP
**40nt2_R**	(Provirus)	CCTGTTTGGCCCATATTCAG	
**UBC E1 L**	Ubiquitin C	CAGCCGTATATCTTCCCAGACT	qPCR
**UBC E2 R**	(UbC)	CTCAGAGGGATGCCAGTAATCTA	
**TRIM28 E4 L**	Trim28	CCTCGTGCTGTTCTGTGAGA	qPCR
**TRIM28 E4/5 R**	E4/5	TGGTACTGATGGTCCTTGTGA	
**SMARCAD1 E11/12 F**	Smarcad1	AGACGAAATGGGCCTAGGAA	qPCR
**SMARCAD1 E12 R**	E11/12	GTGGAGGCTGGAACAACAAT	
**SMARCAD1 E13**	Smarcad1	TCCACCATAGATAACTGGTTAAGAGA	qPCR
**SMARCAD1 E13/14**	E13/14	TGAGAACCATAGTAACAGAGCACAT	
**H3.3A E1/2 F**	H3.3a	CAGCGCCGCCTCTCGCTTG	qPCR
**H3.3A E2 R**	Exon 1-2	CAGTCTGCTTTGTACGAGCCATGGTA	
**H3f3B-E2-E3-F**	H3.3b	CCAAGGCGGCTCGGAAAAGC	qPCR
**H3f3B-E3-R**	Exon 2-3	GGTAACGACGGATCTCTCTCAGA	
**HIRA E7 F**	HIRA	AACTCTGAGAGGTCATTCTG	qPCR
**HIRA E9 R**	Exon 7-9	CACAACAGTCACAGCTTTCC	
**DAXX_E3/4**	DAXX	CTATAGGCCAGGCGTTGACC	qPCR
**DAXX_E4**	Exon 3-4	GCCAAGGTTCGATTTTCCCG	
**shRNA Forward**	pLKO.1 Multiple	ATCGTTTCAGACCCACCTCC	Cloning
**shRNA Reverse**	cloning site	CAGGGCTGCCTTGGAAAAG	

Supplementary Table S2: Antibodies used in this study.

Species in which antibody was produced	Target	Clonality	Stock concentration	Dilution of stock for WB	Source, cat number	Use in lab
Mouse	Trim28	Monoclonal	1mg/ml	N/A	Abcam, ab22553	ChIP
Rabbit	HA tag	Polyclonal	1mg/ml	1:1000	Abcam, ab9110	ChIP & WB
Rabbit	Smarcad1	Polyclonal	N/A	1:1000	Sigma, HPA016737	ChIP & WB
Rabbit	GAPDH	Polyclonal	200 µg/ml	1:1000	Santa Cruz, sc-25778	WB
Rabbit	IgG	Polyclonal	N/A	1:100	Santa Cruz, sc-2027	ChIP & WB
